# Vegetable Grafting: A Toolbox for Securing Yield Stability under Multiple Stress Conditions

**DOI:** 10.3389/fpls.2017.02255

**Published:** 2018-01-12

**Authors:** Youssef Rouphael, Marios C. Kyriacou, Giuseppe Colla

**Affiliations:** ^1^Department of Agricultural Sciences, University of Naples Federico II, Portici, Italy; ^2^Department of Vegetable Crops, Agricultural Research Institute, Nicosia, Cyprus; ^3^Department of Agricultural and Forestry Sciences, University of Tuscia, Viterbo, Italy

**Keywords:** breeding, interacting stressors, root system architecture, rootstocks, signaling pathways, yield gap

## Bridging the yield gap: a question of sustainability

The average yield harvested worldwide amounts to roughly half of the yield obtained potentially given optimal conditions for crop growth. The difference between actual and potential yield, known as the yield gap (Alexandratos and Bruinsma, 2012), ranges widely worldwide and may reach up to 60–70% in developing countries with reference to agronomic and vegetable crops (Alexandratos and Bruinsma, 2012). Biotic factors, including soil-borne pathogens and nematodes, account for roughly one third of the yield gap, the rest attributed to abiotic factors including salinity, drought, flooding, waterlogging, heavy metal contamination, suboptimal temperatures and nutrient deficiencies, and toxicities (Savvas et al., 2010; Schwarz et al., 2010; Peleg et al., 2011; Spiertz, 2012). Under climate change forecasts, the pressure of biotic/abiotic stressors on yield is expected to rise and challenge further global food security. Food demand worldwide is expected to rise by 70–100% in the years up to 2050 and closing the yield gap relies on the triptych of breeding higher yielding cultivars, optimizing resource use efficiency, and reducing postharvest waste (WHO, 2016).

Raising the yield potential solely through breeding faces several shortcomings, as it gears for high input production systems of questionable sustainability and inevitably widens the yield gap under suboptimal conditions. Moreover, breeding for high yielding cultivars is implicated in the diachronic decline of sensory and functional quality of fresh horticultural products, undermining efforts in improving human nutrition through the intake of bioactive foods (Causse et al., 2002; Klee and Tieman, 2013; Kyriacou and Rouphael, in press). Yield stability in agroecosystems faced with complex biotic/abiotic stressors is a critical component of breeding increasingly factored in by climate change. It might be argued that improving yield stability against multiple biotic/abiotic stressors must be at the core of future efforts in addressing global food security, complimented by optimized and sustainable use of production resources (Atkinson and Urwin, 2012).

Compiling desirable traits against multiple stressors while avoiding undesirable combinatorial or pleiotropic effects hampers breeding efforts. In herbaceous annual crops, selecting desirable characters has been eased by advances on grafting, characterized as a surgical alternative to breeding through the coupling of two independent genotypes selected respectively for desirable root and shoot traits (Albacete et al., 2015; Koevoets et al., 2016). In the case of vegetable crops, grafting was essentially rediscovered in the past two decades and expanded on unprecedented scale as an effective and sustainable alternative to soil sterilization by means of chlorofluorocarbon fumigants (Kyriacou et al., 2017). Advances in our understanding of rootstock mediated effects on scion performance have furthered the scope of grafting toward cultivation under adverse environments. Grafting may facilitate the exploitation of root physiological stress tolerance reserved in wild genetic resources for cultivating vegetable crops under stress conferred by conditions of salinity (Colla et al., 2010), nutrient deficiency or toxicity (Savvas et al., 2010; Colla et al., 2011), water shortage (Rouphael et al., 2008), organic pollutants (Schwarz et al., 2010), and alkalinity (Borgognone et al., 2013). Under conditions of stress impinged by soil abiotic and biotic factors, rootstock influence on plant performance outweighs that of the scion (Koevoets et al., 2016), moreover there is mounting evidence of rootstock effects on the configuration of fruit sensory and phytochemical profiles (Kyriacou et al., 2017). Undoubtedly, the growing application of vegetable grafting has been propelled by pressing demand for such root characteristics as resistance to soilborne pathogens, (Louws et al., 2010), tolerance for abiotic stressors (Schwarz et al., 2010; Kumar et al., 2015; Rouphael et al., 2016) and yield proliferation (Lee et al., 2010). Notwithstanding recent advances in our understanding of rootstock-scion relations under the above conditions of stress, combined stress factors have not yet received due attention although grafting is primarily practiced under open field conditions of multiple stress. Understanding crop response to multiple stressors warrants research firstly under controlled environments, where the interaction between multiple stressors can be elucidated, before verification of results under open field conditions.

## Rootstock-mediated tolerance to biotic and abiotic stressors

Vegetable crops grown under greenhouse and especially open-field conditions are faced with multiple biotic and abiotic constraints reiterated above, which hamper crop growth and productivity (Lee et al., 2010). The effectiveness of grafting in imparting tolerance to vegetable crops against abiotic and biotic stressors has been attributed to several improved traits of grafted plants: (i) more vigorous root system apparatus, (ii) improved water and nutrient uptake, (iii) enhanced photosynthetic efficiency and water relations, (iv) stronger antioxidative defense system, (v) heightened hormonal signaling, and (vi) large and long-distance movement of mRNAs, small RNAs and proteins (Albacete et al., 2015; Warschefsky et al., 2016; Kumar et al., 2017). These mechanisms influence both root and shoot functioning, and the interconnectedness of the factors implicated (rootstock, scion and environment) hide singular contributions to phenotypic adaptation (Warschefsky et al., 2016).

Assuming that root is the first tissue sensing stressful soil conditions, potential rootstocks are selected based on traits inherent to the root apparatus itself (Nawaz et al., 2016). The advantageous root system architecture (higher biomass) of selected rootstocks enables better performance against soilborne pathogens and suboptimal abiotic conditions, through improved uptake of water and macro/micronutrients (Nawaz et al., 2016; Cohen et al., 2017). Much remains to be understood regarding the contributions of the below and aboveground plant parts to induced systemic defense, however it has been demonstrated that selected rootstocks impact the uptake and transport of nutrients to the scion by deploying mechanisms (e.g., higher expression levels of ion transporters), that can modulate plant growth and productivity under conditions of stress (Albacete et al., 2015). Scion/rootstock combinations may command a shift in the root microbiome (i.e., endophytic fungi and Plant Growth Promoting Rhizobacteria). Microbial communities in the rhizosphere of the rootstock can influence the composition of root exudates (i.e., sugars, amino, and organic acids) and the uptake of micronutrients, they can generate hormones that foster tolerance to abiotic stresses and also suppress soilborne and foliar pathogens and pests (Liu et al., 2009; Louws et al., 2010). Recently, Cohen et al. (2017) reported that disease suppressive effects in grafted plants could be interpreted in terms of direct resistance of the rootstock as well as to the induced resistance of the scion. Among the mechanisms employed by tolerant rootstocks against biotic and abiotic stressors is enhanced antioxidant defense (Louws et al., 2010; Kumar et al., 2017), achieved by activating antioxidant enzymes (i.e., APX, CAT, SOD) as well as through non-enzymatic antioxidants (i.e., ascorbate, carotenoids, glutathione, and tocopherols) to scavenge Reactive Oxygen Species (ROS), thereby shield cells from oxidative wear (Colla et al., 2010, 2013; Kumar et al., 2015).

Regulation of hormonal synthesis and their precursors has been also proposed as a mode of action by which rootstocks mediate plant growth and modulate stomatal conductance (abscisic acid) and leaf senescence (cytokinins) under stress conditions (Albacete et al., 2009, 2015). In a series of experiments, Albacete et al. (2008, 2009, 2010) demonstrated that the performance of commercial tomato cultivars grafted onto *S. lycopersicum* L. × *S. cheesmaniae* L. Riley hybrids and grown under salt stress conditions was highly correlated to leaf xylem *trans*-zeatin concentration as well as to indole-3-acetic acid and ABA. In line with the previous studies, grafting wild-type plants onto tomato rootstock expressing *isopenthyl adenosine transferase* gene promoting cytokinin synthesis caused significant increase in yield and trans-zeatin concentration in comparison with salinized wild-type self-grafts (Ghanem et al., 2011). Augmented root to shoot transfer of cytokinins enhanced crop productivity of salinized tomato by reducing flower abortion, increasing fruit size and stimulating sink metabolism (Albacete et al., 2014). Furthermore, grafting itself generates differential expression of microRNAs when self-grafted watermelon was compared to plants grafted onto rootstocks of squash and bottle gourd (Liu et al., 2013). The movement of mRNA through the phloem from the rootstock to the scion could be considered of high relevance to several biological and metabolic processes able to regulate plant growth and development moreover adaptation to environmental conditions (Kyriacou et al., 2017). While grafting seemingly introduces additional complexity to our understanding of the mechanisms implicated in plant response to stressors, it also opens a gateway to confronting multiple stressors through independent selection of desirable root and shoot traits.

## Confronting the combinatorial effects of multiple stressors

As climatic extremities pose a serious threat for global food security, there is a growing acknowledgment of the importance of crop resilience and yield stability. Crop productivity worldwide is reduced by more than half due to environmental stress factors, such as heat, drought, salinity, flood, and cold (Wang et al., 2003), hence it is becoming evident that research attention must be turned to unraveling plant response to interacting multiple stressors (Atkinson and Urwin, 2012). Response to single stressors, administered under controlled environment studies, is mediated by unidirectional hormonal signal transduction pathways which may allow for convenient extrapolation. Under open field conditions however, genotypes developed for tolerance against individual stressors are confronted with an interactive network of multiple stressors. Such interactions may involve multiple abiotic and biotic stressors underscored by synergistic negative or positive effects that exceed the additive effect of the individual stressors involved (Rizhsky et al., 2004; Mittler, 2006; Collins et al., 2008). For instance, several studies have highlighted that the combinatorial stress effects of heat and drought on growth and yield of rainfed crops exceeded the sum of the individual stress effects (Jagtap et al., 1998; Jiang and Huang, 2001; Wang and Huang, 2004). Indicatively, Mittler (2006) reported that total losses in the US 1980-2004 agricultural production attributed to drought alone were estimated at US$20 billion, however when combined with heat stress losses exceeded US$120 billion, highlighting how the presence of a secondary stress factor can multiply another's effect. Likewise, Prasad et al. (2011) reported that the combinatorial stress effects of heat and drought on spring wheat growth and yield were greater than the additive effects of the individual stressors. It is evident that securing yield stability calls for developing plant genotypes possessing tolerance traits to multiple stressors, particularly drought, salinity and heat that are the ones forecasted to escalate most according to climate change models (Suzuki et al., 2014).

Plant response to combined stressors is mediated by interacting metabolic and signaling pathways, including transcription factors, photosynthetic activity, ROS scavenging activity, hormonal signaling, and synthesis of osmolytes, and phenyl propanoid defense compounds, as reviewed extensively by Atkinson and Urwin (2012), and Suzuki et al. (2014). Although much remains unknown with respect to the mechanisms imparting stress tolerance, it is evident that stressors compromise plant photosynthetic capacity and that plant response to stress conditions commands for an optimal distribution of resources between growth, reproduction and tolerance mechanisms. Of particular relevance to vegetable crops is also the higher sensitivity to environmental stressors exhibited by reproductive than vegetative growth. It is therefore critical to stack traits of tolerance onto traits of high photosynthetic capacity, which might require strikingly disparate traits for the root system, the vegetative and reproductive organs. In the case of vegetable crops, a short-cut to such a demanding combination of traits might be facilitated through grafting.

## Challenges ahead

Bridging the yield gap is increasingly evolving into a question of yield sustainability under multiple stressors exacerbated by the ensuing climate change. Improving yield stability through breeding requires traits of tolerance against interacting multiple stress conditions, while cases of positive interactions among stressors may be exploited to enhance the nutritional/bioactive value of foods. Vegetable grafting offers a unique alternative to conventional and transgenic breeding for compiling tolerance traits and improving yield potential, yield stability and even product quality. To achieve these goals, future research on grafting must address the core issue of plant response to multiple stressors, encompassing reciprocal work under field and controlled environments. In the words of David Morgan, Syngenta's Global Head of Vegetables and Specialties, during the International Tomato Grafting Conference in Almeria-Spain (January 2015): “*Rootstock technologies have become a key tool to providing not only properties against biotic root stress but also abiotic stress protection against temperature fluctuations or salinity. Definitely, rootstock techniques are increasing crop sustainability in terms of productivity, consistency and quality*.”

## Author contributions

YR, MK and GC had the original idea. YR and MK: Bridging the yield gap: a question of sustainability. YR and GC rootstock-mediated tolerance to biotic and abiotic stressors. MK Confronting the combinatorial effects of multiple stressors. All authors: Challenges ahead. MK and YR contributed significantly to improve the final version of the article.

### Conflict of interest statement

The authors declare that the research was conducted in the absence of any commercial or financial relationships that could be construed as a potential conflict of interest.
